# On Etherization

**Published:** 1848-06

**Authors:** 

**Affiliations:** Washington, D. C.


### MEDICAL EXAMINER,

AND

### RECORD OF MEDICAL SCIENCE.

NEW SERIES.—No. XLII.—JUNE, 1848.

### ORIGINAL COMMUNICATIONS.

*On Etherization.* By Professor Lindsly, of Washington, D. C.

To the Editor of the Medical Examiner.

Having observed, in several papers, notices of the Report which I presented at the late meeting of the American Medical Association in behalf of the Committee on Obstetrics, that are erroneous in various respects, I beg leave through your valuable journal to offer a few remarks on etherization, in which some of these errors will be corrected.

It has always been very remote from my intention, to take an ultra or partizan stand in favour of etherization in midwifery. I believe, in the very great majority of cases, no interference with the natural progress of labour is necessary or justifiable, but I also believe that there are cases where it is proper for the practitioner to resort to a remedy, which is confessedly efficient in relieving pain, and which I have no doubt is, with due caution, entirely safe. And I regret to see physicians of high standing in the community, not only condemn without trial, but take the lead in denouncing, means, of which they are *experimentally* ignorant, thus reversing the sound advice of Hunter to Jenner—" Do not think, but try,"—for these gentlemen say by their actions, " we will think (and condemn,) but we will not try."

Those who object to the TRIAL of chloroform in midwifery as unsafe, seem to forget that it is possible to make a trial of it without producing the *full* anæsthetic effect. I contend, and I know it by personal observation, that an effect very far short of complete anæsthesia, will give very great relief, by allaying pain, and especially by soothing that nervous excitability, which is so distressing to many parturient women. The inhalation of ten or twenty drops of chloroform will often accomplish this, and I do not believe a patient can be found who could not inhale this quantity with perfect safety, especially if the handkerchief or sponge be occasionally removed (for a moment) from the mouth or nostrils, so that atmospheric air alone may be inspired. There can be no doubt, that chloroform, like all other narcotics, *can* be given in doses that are unquestionably safe, and that these smaller doses may be of great benefit, without giving entire relief, just as opium or any other anodyne may soothe pain, without wholly removing it. *Complete* insensibility cannot be produced by opium, without giving it in dangerous quantities, and yet no one pretends for a moment that this is any reason, why it should not be employed in quantities that are safe, for the purpose of affording *partial* relief. If we should admit, therefore, for the sake of argument, that chloroform cannot be safely given so as to produce complete anæsthesia, there still remains the same reason for prescribing it, as leads us to the use of other narcotics, viz : that it can be given with perfect safety, so as to relieve pain, without causing insensibility. Its safety (given in this way) and efficiency being admitted, it unquestionably possesses three most important advantages over opium : it produces its effect almost instantaneously; it does not retard, but rather hastens the progress of the labour ; and it causes no ulterior bad results.

The important practical doctrine which I wish to inculcate is this : that sufficient evidence has now been adduced in favour of etherization in midwifery practice—it having been employed in probably two thousand cases without a single fatal result—to render it the duty of the profession to give it farther trial, to experiment with it, cautiously and judiciously, in order to see if we cannot finally arrive at general laws and principles, which will enable us to administer it without danger or apprehension.

*Washington, May 11th,* 1848.

